# Only when the boat has started sinking: A maternal death in rural north India^☆^

**DOI:** 10.1016/j.socscimed.2010.05.002

**Published:** 2010-11

**Authors:** Patricia Jeffery, Roger Jeffery

**Affiliations:** School of Social and Political Science, University of Edinburgh, Chrystal Macmillan Building, Edinburgh EH8 9LD, United Kingdom

**Keywords:** India, Maternal mortality, Institutional delivery, National rural health mission, Janani Suraksha Yojana (JSY), Women, Muslims

## Abstract

This paper uses a close reading of villagers’ responses to the death in childbirth of a Muslim woman to raise questions about India’s current policy emphasis on institutional delivery as a means of reducing maternal mortality. After introducing the context and methods of our research, we describe recent policy interventions related to maternal health, including the National Rural Health Mission established in 2005. We then outline villagers’ commentaries on the specific maternal death, focusing on the costs to women’s health (and sometimes life) of high fertility; the lack of care available from rural government facilities and staff and the preference for delivering at home with the aid of local practitioners; the financial constraints that make people hesitate to seek medical treatment; and the high costs of private treatment and the poor treatment experienced in government facilities. Our core argument is that government health care provision in rural Uttar Pradesh is embedded in a moral universe characterised by widespread and long-term mistrust of state services and that encouraging institutional deliveries without addressing the perceptions of potential service users is a seriously flawed approach to reducing maternal mortality. The paper draws primarily on ethnographic research funded by the Wellcome Trust during 2002–2005, in a Muslim village in rural Bijnor district (in north-western Uttar Pradesh).

*Anjam:* Children are born in the village inside houses. Women are not taken to the town. Only ‘when the boat has started sinking’ [*jab kashtī dūbne lagī*, i.e. at the very last moment] do they run off to town. (Authors’ fieldnotes 19 February 2003; all personal names used in the paper are pseudonyms.).

In 1982 we had attended the marriage of Razia to Rashid, a young Muslim man from Jhakri, one of our study villages in Uttar Pradesh [UP], north India. By 2002, Razia had had one stillborn son and there were nine living children, the youngest a toddler. In late summer 2002, Razia was pregnant again but matters went badly wrong: after a long labour, she was taken to the government women’s hospital in Bijnor town, where she and the baby had both died shortly after admission.

This paper juxtaposes villagers’ commentaries on Razia’s tragic experience with policy initiatives aimed at combating high levels of maternal mortality. After describing the context and methods of our research, we describe recent policy interventions related to maternal health, including the National Rural Health Mission [NRHM] established in 2005, under which women are encouraged to deliver their babies in institutions rather than at home. Drawing on our long-term fieldwork, we then present the villagers’ accounts of Razia’s death and their perceptions of the health care services available to them.

The success of policy initiatives such as NHRM certainly requires that systemic failures of provision are remedied—but it is also vital to foreground and address the perceptions of those supposedly being served by such initiatives. Our core argument is that the advocacy of institutional deliveries is not entering a morally neutral terrain. Rather, government health care provision in rural UP is embedded in a moral universe characterised by widespread and long-term mistrust of state services. Clearly, supply-side problems—lack of equipment or technically skilled staff—need to be dealt with if ‘safe’ delivery is to become the norm. Indeed, villagers often point to such limitations in the government health services in general. But failing to respond to complaints about government health staff—about illegal demands for payment and demeaning and discriminatory dealings with patients, for instance—make it unlikely that women will readily opt for delivery in a government institution. Evidence from elsewhere in UP and India more generally indicates that the villagers in Jhakri are by no means unusual in their critical stances—whilst early evaluations of NRHM suggest that extortion and rudeness continue to be experienced by women seeking institutional deliveries.

## Research context and methods

This paper draws on our long-term ethnographic work in rural Bijnor district, in north-western UP. In 2001, Bijnor town (district headquarters, population about 100,000) had two government hospitals, one general and one for women, plus some 20 private nursing homes offering obstetric services. The district’s population was about 3.1 million, of whom about 1.3 million were Muslims; about 34 per cent of the rural population is Muslim (Census of India, 2001). In UP, Muslims comprise about 18 per cent of the state’s overall population and are a socially and economically marginalised population (Sachar, 2006).

In 1982–1983, we were based in two villages to which we returned several times between 1982 and 2002–2005. They are about 5 km by metalled road from Bijnor town. Dharmnagri had a Hindu and Dalit/ex-Untouchable population of 1125 in 2002; there is a government Additional Primary Health Center (PHC) on its periphery. About 500 m away is Jhakri, the main focus of this paper. Jhakri is a Muslim village with a population of around 665 in 2002, mainly Sheikhs—a middle ranking caste-like group—along with a handful of Teli and Julaha households. A few adult women in Jhakri were both born and married there, but over 92 per cent of the married women were born elsewhere, mainly within the district.

Bijnor district shares unfavourable social and demographic indicators with the rest of UP, but fares relatively well on indicators of economic development because Green Revolution packages introduced in the mid-1960s transformed agriculture. The economic inequalities in Jhakri are not as stark as in many other Bijnor villages because landholdings are generally small (rarely more than 0.4–0.8 ha). In Jhakri, Sheikhs own most of the village land, but few can rely wholly on agriculture. Villagers with little or no land seek other employment (e.g. plying cycle rickshaws or working as motor mechanics in Bijnor town, or agricultural labour).

In 1982–1983, our research focused on the social organisation of childbearing. We collected quantitative data (including household censuses and maternity histories for all the ever-married women in the two villages) and qualitative data (ethnographic observations, informal conversations, and especially semi-structured interviews with twenty key informant couples in each village, covering childbearing, family planning and health care, as well as household politics, marriage arrangement, dowry, land, employment, education, migration). We also interviewed health care providers, including traditional birth attendants (*dāī*) and untrained practitioners (P. Jeffery, Jeffery, & Lyon, 1989). During subsequent fieldwork visits, we updated the village data, culminating in the 2002–2005 research, which explored the original key informants’ trajectories, particularly in relation to health care, childbearing, family building and sustainable livelihoods in the context of social and economic change since the early 1980s. This research also entailed interviews with *dāīs*, and rural and urban practitioners, on which we do not draw directly here.

We have both been involved in planning and conducting the research, although Patricia did almost all the data collection during 2002–2005. We both speak Hindi and conducted the interviews ourselves. Ethnographic research, however, is an inherently dialogic process that entails taking people’s concerns and questions seriously. Our interviews often became discussions in which bystanders—husbands, neighbours—joined. Tape-recording was impractical and we have employed women from Bijnor town as research assistants, primarily as scribes although they also contributed to the discussions. The assistants took detailed notes which they wrote up in Hindi immediately afterwards. We checked the assistants’ reports for completeness and clarified ambiguities of interpretation before translating the reports into English. These typed fieldnotes comprise some 4500 single-spaced pages and they form the basis for the qualitative data analysis. We indexed the data from the early fieldwork manually onto index cards; for the most recent data, we used the electronic qualitative data analysis package Atlas.ti. We constructed nested index categories, with broad categories sub-divided into finer-grain categories, as well as indexing named individuals. The broad categories included agricultural production and household organisation, as well as health-related issues (such as childbearing, family planning, government health services, government and private medical practitioners). We indexed every passage in the fieldnotes; where discussions touched on several topics we indexed them under each appropriate category. Our close familiarity with the data enabled us to conduct searches of the indexed material to develop emergent analytical categories, look for counter-examples and (for selected topics) tabulate the qualitative data. This paper draws on such consolidated information about our discussions with villagers on the topics that we address below. The quotations in this paper are from our translations, selected to highlight either general views or the range of views on a particular issue.

The research received ethical approval from the research ethics committee of the School of Social and Political Science (University of Edinburgh) and we have followed the ethical guidelines of the British Sociological Association throughout our research (see http://www.britsoc.co.uk/equality/Statement+Ethical+Practice.htm). Our guarantees of confidentiality and anonymity and our frequent visits since 1982 have been vital in building the trust that is reflected in numerous conversations with villagers who have (for example) criticised government health staff without fear of recriminations.

This paper focuses on villagers’ perceptions and experiences of seeking health care. It draws mainly on discussions in Jhakri during 2002–2005, but is informed by our earlier fieldwork in the two villages. Updating the maternity histories often resulted in wide-ranging discussions about institutional deliveries, family planning and child immunisation, for instance, including unsolicited comments about Razia’s death, which had occurred shortly before Patricia’s arrival in September 2002. Villagers certainly knew of other women who had died in childbirth—but Razia’s was the only maternal death in either Jhakri or Dharmnagri during the 20 years we had worked there (although some women reported what they considered ‘near-misses’ that were averted by emergency hospital admissions) (P. Jeffery & Jeffery, 2008). Moreover, Jhakri is a compact village and news spreads rapidly. It is unsurprising that Razia’s experiences became such a talking point, often referred to by villagers wanting to emphasise points germane to their own childbearing experiences or health care issues in general. Before examining villagers’ perceptions of Razia’s death, however, we outline the wider background in UP.

## Maternal mortality in Uttar Pradesh

North India (including UP) has high maternal mortality ratios: MMRs for UP (including Uttaranchal) were estimated to be about 900–950 in the early 1980s and 700–750 by 1990 (Bhat, 2002); by 2001–2003, they were 517 (95 per cent CI 461–573) (Registrar-General India, 2006) and by 2004–2006, 440 (95 per cent CI 384–495) (Registrar-General India, 2009). Vital events registration is unreliable and these estimates are derived from special surveys. With some 5 million births in UP annually, there are at least 25,000 and possibly as many as 35,000 maternal deaths per annum.

Maternal mortality reflects several interlinked causes: medical conditions directly associated with childbearing (e.g. haemorrhage, sepsis, eclampsia, obstructed labour); other medical conditions (e.g. malaria, heart disease); and socio-economic factors such as patterns of gender politics that result in early marriage and high fertility, and compromise access to nutrition, health care and contraception, especially amongst the poor, villagers and marginalised communities. Indian government policies have done little to address the social determinants of maternal mortality: rather, as elsewhere in the global South, the approach has typically been top-down and narrowly medical in orientation (Sen, Govender, & Cottingham, 2007; Sen, Őstlin, & George, 2007).

In UP, Government health services have been dogged by long-term inadequacies that bias access to health care in general, to the detriment of those very women most at risk in childbearing. Nearly 80 per cent of UP’s population lives in rural areas, yet government facilities reflect a marked urban bias and rural facilities are generally located only in larger villages. Thus many patients cannot easily access medical attention. Further, rural facilities often lack basic equipment and their full component of qualified medical staff (whether because of unfilled posts or staff absenteeism) (Devarajan & Shah, 2004: p. 910; Infrastructure Division, 2006; IIPS, 2003; Planning Commission, 2002: Vol. 2, pp. 86–87; Sen, Iyer, & George, 2002: p. 293; Vora et al., 2009). Restrictions on who may administer anaesthesia (for instance) further reduce villagers’ access to emergency obstetric care (Mavalankar & Sriram, 2009). Budgetary cuts imposed through World Bank loans in the 1990s and early 2000s further undermined the UP state’s already weak capacity to finance its health care sector. Meanwhile, the burgeoning private sector now dominates curative medical care—often requiring poor villagers to make out-of-pocket expenditures that they can ill afford (P. Jeffery & Jeffery, 2008).

Until the early 2000s, antenatal clinic-based monitoring was the main means of addressing maternal mortality. Yet recent National Family Health Survey (NFHS) data from UP indicate that only about 26 per cent of pregnant women made three antenatal visits and only about 8 per cent of all pregnant women received iron-folic acid for six months before their most recent delivery (Government of India, 2007). Moreover, women from poor and marginalised communities tended to have least antenatal care, and that, too, of generally low technical and interpersonal quality (CRR, 2008: pp. 15–17; Pallikadavath, Foss, & Stones, 2004; Rani, Bonu, & Harvey, 2008).

In 2005—three years after Razia’s death—India’s National Rural Health Mission (NRHM) was launched. The NRHM reflects the changing priorities in global discourse towards the conditions of delivery and the promotion of ‘essential obstetric care’, which includes ‘skilled’ birth attendants (SBAs) and institutional deliveries, buttressed by comprehensive (or emergency) obstetric care (operating facilities, blood banks etc.) and effective referral systems (e.g. Berer, 2007; Berer & Ravindran, 1999; Freedman et al., 2005: especially pp. 77–94, 132–135; Maine, 1999; WHO, 1999). Various NRHM provisions were intended to counter the ‘three phases of delay’ (delay in seeking treatment, in reaching a facility and in obtaining care once there) (Thaddeus & Maine, 1990). Incentive payments under Janani Suraksha Yojana (JSY, or Mother Protection Programme) should encourage women to deliver in institutions (Ministry of Health and Family Welfare, 2006); referral systems should guarantee their timely arrival; and upgraded facilities and trained staff should ensure prompt and safe delivery care. Village women trained as Accredited Social Health Activists (ASHAs) and integrated with existing health staff would provide antenatal, intrapartum and postnatal care (Rajalakshmi, 2005), motivate women to seek institutional deliveries and accompany labouring women to the relevant facilities.

Preliminary evaluations of NRHM indicate serious supply-side limitations. Overall, spending of earmarked funds has progressed very slowly (USAID, 2007), so the health care system cannot respond adequately to the NRHM demands. As one of the eight Empowered Action Group (or ‘low-performing’) states, UP was allocated additional NRHM funding—but the programme has been 30–40 per cent under-spent in all its first three years (HRW, 2009: p. 27). ASHA training programmes are seriously behind schedule and critics question the capacity of referral systems and of Government institutions to guarantee the comprehensive and high quality care necessary to ensure safe outcomes (CRR, 2008; CHSJ, 2007; HRW, 2009). Moreover, the NRHM’s progress is evaluated primarily via increases in institutional deliveries: bizarrely, there is no mechanism to ensure that maternal deaths are systematically recorded (HRW, 2009: p. 12ff.).

## Villagers’ accounts of Razia’s death

In addition to system failures, however, it is also vital to understand the demand side: villagers’ perceptions of local health care options. Strikingly, people’s commentaries on Razia’s death were not differentiated by age, marital status or economic position. Their accounts often straddled issues that we have separated here for ease of presentation. They were, of course, given several months after the event and villagers were often vague about medical details—but for our argument, the ‘truth-value’ of these accounts is less important than what they convey about villagers’ interpretations of the tragedy, their attributions of causality and blame and the likely effects on future decision-making.

### Fertility and maternal health

Our fieldnotes from 1982 onwards contain numerous comments made by women on the risks of childbirth and the damage to health of undergoing numerous pregnancies. One woman asserted in 1982, ‘women become old bearing children. They can’t keep their strength. Their spirit drains away’ (P. Jeffery et al., 1989: p. 172, see also pp. 167–175). Twenty years later the refrain was much the same: during her twelfth pregnancy, another woman said:

There are 10–10, 12–12 children. You can neither look after yourself nor the children. You can’t flourish. Just as you’ve cared for one, another is born. The ‘vile hardship’ [*gandī āfat*] continues. How can a person flourish? (Authors’ fieldnotes 24 February 2003).

Similar understandings were reflected in commentaries on Razia’s death:

*Talib*: Women here don’t understand that this [childbirth] is women’s death. Just look at Razia. She herself died in giving birth. And behind her, her children are worried and her husband is also worried. She’d had 11–12 children and there was no life in her body. She had become weak. (Authors’ fieldnotes 24 February 2003)

*Mehbuba*: Some children are their mother’s enemy and they take their mother with them. [*Patricia*: How could a small child be its mother’s enemy?] Many children before they are born eat their mother. They are their mother’s enemy.

*Mehbuba’s married daughter*: Razia’s child was also like that. It ate its mother. (Authors’ fieldnotes 4 February 2003)

Nevertheless, contraceptive usage in Jhakri is low. Muslim clerics in India have generally claimed that Islam forbids contraception or at least sterilisation (though the theological basis is controversial and contested). This view is widespread amongst north Indian Muslims and few people in Jhakri think sterilisation is permissible. Talib (quoted above) is exceptional: he responded to his wife’s obstetric crisis by taking her to a private hospital, where she was sterilised at the same time as her stillborn girl was delivered by Caesarean section (see P. Jeffery, Jeffery, & Jeffrey, 2008). By contrast, two Jhakri women discussing Razia’s death insisted they would never use contraception:

*Mumtaz (with 9 living children)*: Listen to what I’m saying! However many children I have at present, if that many more are born in future, even then I wouldn’t take anything to stop having children, or make the gap longer, even if I were to die. In our village, Razia died in childbirth and so I am also thinking that death has to come one day or another. So what’s there to fear about death?

*Akbari (with 7 living children)*: No matter how many children we have, we shan’t take any medicines, for we also have to go to meet Allah. (Authors’ fieldnotes 7 February 2003)

But their bravado was unique. Mumtaz’s sister Mehmuda (with 7 living children) is also married in Jhakri: she complained disgustedly about how her children had been born pell-mell [*tale-ūpar*].

Given the widespread poverty in Jhakri, women’s comparatively high levels of fertility probably compromise their health. But Razia’s last pregnancy was additionally complicated by the baby’s transverse presentation.

### Labouring at home

In rural Bijnor, pregnancy is normally dealt with at home. Few Dharmnagri and Jhakri women attended routine antenatal clinics, despite the proximity of the Dharmnagri PHC. Razia, however, had consulted the Auxiliary Nurse-Midwife (ANM) there late in pregnancy and had an ultrasound performed in a private facility in Bijnor town—not a routine investigation for rural women. There had been concerns well before Razia’s labour began:

*Sabra* (*the trained dāī in Jhakri*): The baby was completely transverse [*ārā*] and I myself told Razia a month before [the birth]. I told her the baby wouldn’t be born at home and would have to be born by operation. [*Patricia*: Did Rashid know that?] Yes, he did. And Razia had also gone to see the ANM and she also told Razia that the baby was transverse. … I told her it wouldn’t be within a *dāī’s* competence. (Authors’ fieldnotes 18 October 2004)

*Jamila*: Razia went to have ultrasound before she had her last baby and the doctor told her that the baby wouldn’t be born at home … and that she should come to hospital when the time was due. … [*Patricia*: Had Razia told Rashid and the other people of the house?] Yes, everyone knew that the baby couldn’t be born at home. And that the doctor had also said the baby was in danger. (Authors’ fieldnotes 9 February 2004)

Villagers in Jhakri and Dharmnagri alike were inclined to think that private doctors’ recommendations for institutional deliveries and other interventions were motivated more by thoughts of profit than medical necessity (see P. Jeffery & Jeffery, 2008). Several women reported being warned that they should deliver in an institution, but most stayed at home, and most delivered there successfully. Razia, then, was not remarkable in remaining at home despite warnings about the dangers. Sabra (the trained *dāī*) said that had she not been visiting her brothers in another village, she would have recommended that Razia go to hospital:

Razia was having pains for many days but she didn’t tell anyone. I wasn’t in the village and the [untrained] *dāī* had come from Chandpuri [a nearby village], but she didn’t say that the case was not within her competence. So when the baby’s arm appeared outside, only then did they take her [Razia] to hospital. (Authors’ fieldnotes 18 October 2004)

The ANM posted at the Dharmnagri PHC was present only during normal working hours, however, and then only when her duties did not take her away from the PHC compound. She conducted more deliveries in a small private clinic in her home in Bijnor town than at the PHC. The PHC doctor played no part in obstetric cases. Villagers generally responded to these gaps in provision by calling upon local untrained practitioners. In Razia’s case, first, an untrained *dāī* was called. Strong contractions continued through one night and the following day. The next evening a ‘doctor’ was called—one of several local rural medical practitioners (RMP), men generally without formal training who prescribe remedies from their roadside kiosks. RMPs play a problematic role in childbirth by administering intramuscular injections of oxytocin to augment contractions (P. Jeffery, Das, Dasgupta, & Jeffery, 2007). By the early 2000s, almost 50 per cent of the home deliveries in Dharmnagri and Jhakri featured an RMP and at least one oxytocin injection. These men rarely examined a labouring woman to assess the baby’s presentation: they relied on the *dāī’s* assessment. About the RMP who attended Razia, Qudsia commented ‘he did not tell them that it was not within his competence’ (Authors’ fieldnotes 17 January 2003). Razia’s attendants deemed her situation serious only when the baby’s arm appeared. With no one at the PHC responsible for delivering the baby or referring her elsewhere, she was taken to the government women’s hospital in Bijnor town on a tractor-trolley requisitioned from a neighbour.

### Financial constraints

Rashid’s father had a little land which he had not yet divided amongst his sons. Consequently, Rashid depended on plying a cycle rickshaw in Bijnor town and on various kinds of irregular employment. Villagers were far more vocal about Rashid’s poverty than about the decision not to take Razia to hospital at the onset of labour, though interpretations of the decision-making in Razia’s home differed.

Most commentaries emphasised that Rashid and his family were not culpable—rather, Rashid’s poverty had made him hesitate to take Razia to hospital. As it was, Rashid was struggling financially. One woman explained, ‘Only when he comes home in the evening do they cook the food. He brings the vegetables home and they get cooked.’ Talib and his wife Taranam also highlighted financial considerations:

*Talib*: Razia from my village died because of poverty. Only the women of the house were beside her and they had called a *dāī*. But if they’d taken her to town sooner, then she, poor soul, wouldn’t have died.

*Taranam*: Razia died because of lack of money. If there had been money, she would also [i.e. like Taranam herself] have had an operation [Caesarean]. (Author’s fieldnotes 24 February 2003)

Some women, however, said Razia should have been taken to hospital much sooner and that the *dāī* had been suggesting this for some time before Razia’s in-laws agreed. But for Rashid’s hesitation, Razia would still be alive. Some also said that Rashid had been reluctant to go around the village cap-in-hand requesting loans to cover the expenses until it was too late. For instance, when Patricia suggested to Sabra that Rashid had possibly not taken Razia to hospital because he had no money, she retorted:

*Sabra*: Generally people in this village don’t have money. But when there’s some calamity, they go house-to-house collecting money. Everyone makes donations. One man takes the person to hospital and another goes around the village collecting money and comes [to the hospital] later. And meanwhile the patient has been admitted to hospital and the money is given later. If Rashid had told people earlier that he had no money, then the people of the village would have made donations and given him the money, and poor Razia’s life would have been saved. … the people of the village would certainly have done something or other to help. (Authors’ fieldnotes 18 October 2004)

### Private medical care and care at the government hospital

In India as a whole, institutional deliveries have become more common, but the bulk of the increase has been in the private sector (Vora et al., 2009: pp. 187–188). During 1993–2002, home deliveries still accounted for nine out of ten births in Dharmnagri and Jhakri, but 54 labours that began at home had ended in an institution in Bijnor town (out of 620 deliveries in that period). Of these, only two women—one of them Razia—went to the government women’s hospital. The remaining 52 went to private nursing homes.

Almost all the accounts of institutional deliveries indicated how reluctant labouring women and their attendants were to seek hospital admission, even when the labour seemed to be progressing badly. In the midst of an obstetric crisis, villagers would be poised on a moral knife edge: facing condemnation from fellow villagers for profligacy if they rushed to town too soon or accusations of carelessness from neighbours and urban health care providers alike if they left matters until ‘the boat has started sinking’. Razia’s case highlights this dilemma.

Covering the costs of private medical care was not necessarily easy. In the early 2000s, local daily wage rates for agricultural labourers were about Rs70; a rickshaw driver—such as Rashid—might earn about Rs100 per day. In private nursing homes in Bijnor town, episiotomies or forceps deliveries cost around Rs4,000. Caesarean sections cost about Rs15,000–16,000 for the operation alone: in other words, at least 200 days’ of a labourer’s wages. Moreover, with a family member in hospital, income is lost and travelling and other outlays must be added to the medical charges. Few villagers could make regular savings and no one had health insurance. Obtaining bank loans was extremely difficult—especially during an emergency and especially for the poor. Without ready cash, going cap-in-hand to kin and neighbours was precisely what most people had to do. Often, long-term indebtedness ensued (P. Jeffery & Jeffery, 2008, 2010).

Villagers considered the costs of medicines and other payments made in private institutions were generally higher and more variable than those in government institutions (cf. Chakraborty, 2002: Table 10.9; Mishra, 2005: p. 74). People stressed the serious financial implications of funding private institutional deliveries. Nevertheless, the overwhelming inclination was to do so. The main reason for this apparent economic irrationality relates to villagers’ views of the care they receive in government facilities. As elsewhere in India, Government facilities were far from free in practice and villagers considered that expenditures there were less predictable and more subject to extortion than those in private facilities. And crucially, villagers reported being treated rudely and even punitively by government staff, who were also slow to respond to their medical needs (George, Iyer, & Sen, 2005; D. Mavalankar & Reddy, 1996; Singh, Lahiri, & Srivastava, 2004).

Some women claimed Rashid would have taken Razia to a private facility if he had been able to raise the cash: he had gone to the government hospital only because of his anxiety about the costs of a private delivery. But others asserted that Rashid had been at fault in taking Razia to the government hospital. Several women alleged that Razia and her attendants were treated rudely, and especially that Razia had been slapped and criticised for having more children despite being poor.

*Jamila*: Those *dāīs* in the government hospital—the ones who deliver babies—asked Razia how many children she had. I don’t know if Razia said 8 or 9, but on hearing this, the nurse slapped her face saying “you have so many children and even so you are busy making more.” (Authors’ fieldnotes 9 February 2004)

Other women commented that Razia had received dilatory treatment, and suggested this was because Rashid was a poor villager without the ‘contacts’ in the hospital or the money to help expedite the treatment:

*Qudsia’s married daughter*: Their biggest mistake was taking her to the government hospital. They don’t even talk properly to patients there. If they had taken her to a private place, that would have been good.

*Qudsia*: When they took her to the government hospital, the doctor was very angry and said that they had only just brought her now, and she shouted and swore at them a lot. The pains increased even more and the doctor was sometimes telling them to get a prescription, sometimes this, sometimes that for her. Then when she looked at her [Razia] a long time later, the life was ‘leaving her feet’. She died and the baby wasn’t even born. (Authors’ fieldnotes 17 January 2003)

Women alleged not only that the medical staff failed to do their work in a timely and efficient manner, but that Razia was given a poisonous injection either because she had so many children or because Rashid could not pay what was being demanded:

*Hanifa*: Rashid was poor and he also didn’t have any ‘known-recognised’ people either, so he took Razia to the government hospital. We’ve heard that Razia was given an injection and that the *doctornī* [female doctor] had also slapped her once. Everyone says that if she’d been taken to a private hospital she would have survived. She was taken to the government hospital and that was why the poor thing had to ‘wash her hands of her life’. (Authors’ fieldnotes 25 February 2004)

*Mehmuna’s mother*: Running–running they took her to the government hospital. There they were asked which child this was. And the simpleton [*sīdhā-mann ādmī*] told them it was the ninth. So the doctor gave a poisonous injection. It wasn’t that she heated the baby’s arm [*ānch denā*] so that the child would pull its arm inside again. On top of not doing that, she gave a poisonous injection. [*Patricia*: How do you know it was a poisonous injection?] It wasn’t even 5 min after the injection that Razia died.

*Mehmuna*: When someone dies naturally, their colour remains the same. But she became completely dark. Her face became completely black. It’s only from poison that a person turns black. (Authors’ fieldnotes 17 February 2004)

The following discussion with Latifan, her unmarried daughter and a neighbour Firozi captures the complex interplay of considerations upon which villagers reflected. Our research assistant Shaila had asked if Razia was taken to hospital very late:

*Latifan*: Razia was indeed taken late. She’d been having pains for two days and then at night half of the baby’s arm appeared outside and even so they didn’t take her to hospital. The poor thing died because of that.

*Patricia*: Didn’t the *dāī* see her?

*Latifan*: Sabra wasn’t in the village. The poor thing [Razia] died for want of money. If they’d had money, they would have taken her to hospital two days earlier. Town women go to hospital 2–3 days before the birth. They have money, that’s why. They and their child both survive. The poor soul, Razia, died because of poverty.

*Firozi*: If they’d made some money arrangements, they could have got money from anywhere at all, but they didn’t pay any attention. And they got her admitted in the government hospital.

*Patricia*: Would they have taken her to a private hospital if they had money?

*Latifan’s daughter*: Then they would have taken her ‘private’.

*Latifan*: Razia had been having a great deal of pain for 2–3 days and her baby must have died just at the very time when her hand appeared. Then after spending a whole night [at home] they took her to hospital, so then the poor thing could only die. They’re blaming the doctor.

*Shaila*: Who’s blaming the doctor?

*Latifan*: The people of the village are.

*Patricia*: But why wasn’t she taken to hospital earlier? Was it because there would be strange men there and it was a matter of embarrassment, or was it because they were afraid of the expense?

*Latifan*: It was a matter of fearing the expense. If there’s no money and you go to hospital, you have to pay Rs15–20,000 straight away. Villagers don’t possess money.

*Patricia*: We’ve heard that some doctors don’t speak properly to patients or that they discriminate because of money or because people are Muslims—is that the case?

*Latifan*: If you give the doctors their full money, they do their work properly.

*Patricia*: What happens if the full money isn’t there or it comes late?

*Latifan’s daughter*: Then they give a poisonous injection. Razia didn’t have money and so the doctor gave her a poisonous injection.

*Patricia*: Is that true?

*Firozi*: It’s absolutely true. The doctor gave her a poisonous injection and the doctor also gave Razia a slap.

*Latifan*: If Razia had told them she had 2–3 children, then the doctor would have looked after her properly. But Razia told them she had exactly as many as there were and that was why she was slapped. She was told that she has so many children and even so she still has the desire to produce more. If someone is poor or someone is rich, doctors do not show any partiality. They just need the full money. (Authors’ fieldnotes 27 January 2004)

There are several possible explanations for Razia’s sudden death: the injection might have been oxytocin (which resulted in uterine rupture) or an antibiotic (to which she had a severe allergic reaction), or she might have been about to die anyway. But, crucially for our argument, villagers considered it entirely plausible that medical staff had deliberately administered a deadly injection: Razia’s legs became ‘cold’ [lifeless, a sure sign that she was about to die] almost instantly after the injection and when her body was returned to Jhakri for burial the next morning, they saw that it had become ‘blue’ or ‘black’.

### Razia’s experiences in a longer timeframe

These commentaries on Razia’s death echo critiques of government health services that we have heard repeatedly in both Dharmnagri and Jhakri throughout our fieldwork since 1982. People in both villages often criticised the quality of rural services: the poor condition of the facilities, the lack of equipment and medical supplies. Villagers feared being unable to meet government employees’ demands for payments in case they were punished. And they often commented about staff behaviour—whether their absenteeism, lack of incentive to work assiduously or rude and brusque dealings with patients. In 1982–1983 and in 1990–1991, we lived on the Dharmnagri PHC compound and witnessed numerous interactions between government staff and villagers that endorse villagers’ allegations. Sometimes we also accompanied villagers to urban facilities and can confirm the tenor of interactions there. In 1982, for instance, we took a woman in obstructed labour to the government women’s hospital in Bijnor town. Thinking that Patricia was Punjabi, staff there verbally abused her because she insisted that they provide treatment at night. We subsequently observed staff slapping the labouring woman several times when she cried out in pain, and demanding money for administering medications or cleaning around her bed (see P. Jeffery & Jeffery, 2008; P. Jeffery et al., 1989: pp. 114–118). In brief, villagers were often treated in government facilities not simply (if at all) in terms of their medical need, but according to other characteristics, such as dirty or ragged clothing that signalled their poverty or rural background, rustic speech or a bevy of small children in tow (R. Jeffery, Jeffery, & Rao, 2007; Koenig, Foo, & Joshi, 2000).

Another crucial component of villagers’ perceptions of government services is the family planning programme. It was introduced in the 1950s with sterilisation the most strongly advocated contraceptive method. Especially, but not only, during the political Emergency in 1975–1977, staff were set sterilisation targets and the programme was coercive and target-driven, particularly in relation to religious minorities and the poor in the high fertility areas of north India (e.g. Connelly, 2006; Gwatkin, 1979; Vicziany, 1983). Since the mid-1990s, the target-driven approach has declined somewhat, but the rural ‘family welfare’ (i.e. family planning) component of reproductive and child health expenditures received *increased* funding during the 1990s (despite the general trend of health sector cuts) (see Dev & Mooij, 2005: p. 100; Qadeer, 1998) and the family planning programme retains its high profile.

In 1982–1983, with memories of the Emergency still fresh, many villagers in both Dharmnagri and Jhakri initially suspected us of being involved in the sterilisation programme (P. Jeffery & Jeffery, 2010). Gradually, though, female sterilisation has become normalised in Dharmnagri and by 2005 about 30 per cent of the Dharmnagri women under 40 had been sterilised, compared with only three Jhakri women. People in Jhakri—and Muslims in north India more generally—have all too often felt themselves vulnerable to the pressures of the Indian government’s family planning programme (P. Jeffery & Jeffery, 2006: pp. 39–42, pp. 108–116; 2010; P. Jeffery et al., 2008; P. Jeffery et al., 1989: p. 200 ff.; R. Jeffery & Jeffery, 1997). Indeed, not only family planning workers are preoccupied with their fellow-citizens’ fertility: we took Najma to consult an ophthalmologist, whose first questions were how many children she had and whether she had been sterilised (P. Jeffery & Jeffery, 1996: pp. 53–68). More recently, many Muslims in north India—including in Jhakri—have mistrusted the coercive tactics adopted during the polio eradication programme and rumours have been rife that polio vaccine renders children infertile (P. Jeffery & Jeffery, 2010).

There is, then, widespread and lingering mistrust of government health care services, particularly but not only amongst Muslims. Villagers consider government services inadequate in terms of equipment, medicines and staffing, but they also object to being treated in a dilatory, discourteous or greedy fashion by government staff. Village women generally wanted to avoid going to government institutions for their deliveries and preferred to stay at home, reliant on relatively cheap local practitioners (*dāīs* and local male practitioners). For Muslims, the government’s fixation with family planning compounds their lack of faith in government provision. Women in Jhakri were not surprised to hear that Razia—whose name and clothing would have marked her as a Muslim—had been rudely questioned about her children, slapped and chided by hospital staff, and treated in a callous way before being given what they believed was a fatal injection.

## Discussion

Given this history, it is unlikely that there will be heavy demand for institutional deliveries in rural Bijnor. We have not conducted in-depth fieldwork in Jhakri since NRHM began, but indications from Bijnor town and elsewhere in UP support this view. In November 2007, during another research project, we visited the government women’s hospital in Bijnor town. The medical superintendent reported that the labour ward bed-occupancy rate had increased from 10.7 per cent in 2003–2004 (just before she was posted there) to 19.3 per cent in 2006–2007. Annualised figures suggested that there would be almost 300 normal deliveries and nearly 225 Caesarean sections in 2007. Yet some 2500–3000 babies are born in Bijnor district *each day*. In UP as whole, deliveries in institutions (public or private) accounted for 21.4 per cent of all deliveries before NRHM was introduced and 24.5 per cent in 2007–2008. There are wide variations between districts and between rural and urban areas but these figures suggest a general and continuing reluctance—not just among Muslims—to seek institutional deliveries (IIPS, 2009).

A Human Rights Watch report (focusing on UP) and a Center for Reproductive Rights report both evaluate the NRHM from a human rights perspective. They present evidence of systemic failures: lack of equipment, drugs and blood, insufficient accessible staff competent to provide emergency obstetric care (such as Caesarean sections). In other words, institutional deliveries in UP are not necessarily ‘safe’ deliveries (CRR, 2008: p. 18ff.; HRW, 2009: p. 30ff.). Staff corruption and demeaning and discriminatory treatment continue to compromise access for the poor and marginalised. Deliveries in government institutions are supposedly free and the JSY payment should provide cash assistance for the mother and baby. But just 4.7 per cent of women giving birth in UP between April 2006 and the IIPS survey date (November 2007–April 2008) received the JSY payment (IIPS, 2009) (probably partly because most institutional deliveries in UP are in private facilities). Women sometimes reported that staff demanded a share of the JSY payments for services rendered or that payments were made months in arrears; often women were either discharged before the risk of post-partum haemorrhage was past or compelled to remain in the institution longer than they wished in order to receive the JSY payment (CHSJ, 2007: pp. 109–117). Some government staff nurses refused to hand over the baby unless they were paid; others made false claims about women who had delivered at home and took a share of the JSY payment; the targets for institutional deliveries provided an additional incentive for government staff to inflate the numbers (HRW, 2009: pp. 11–12, 51ff.). There is caste-based discrimination and cruel and degrading treatment such as beating, pinching and name-calling (e.g. CRR, 2008: pp. 20–23; HRW, 2009: p. 27, pp. 46–47, p. 57) and indications that the polio and family planning campaigns divert attention from maternal health concerns (HRW, 2009: p. 28, pp. 35–36). In other words, the perceptions of people in Jhakri are echoed in UP more generally.

The Human Rights Watch and Center for Reproductive Rights reports both emphasise that human rights entitlements entail access to health, not just health care (CRR, 2008; HRW, 2009). Clearly NRHM is not expected to tackle the social and economic determinants of women’s vulnerability. But improving access to quality health care should go beyond a focus on facilities, equipment and skilled attendants. Women’s birthing experiences must be not just medically ‘safe’ but also ones in which they are not confronted with staff who embezzle government funds, extort money illegally and treat patients in distress in discriminatory, demeaning and punitive ways. By emphasising the views of people supposedly served by the NRHM, we want to highlight how people’s memories of their past relationships with state services linger and can generate resistance to new policy initiatives among at least some sections of the citizenry. Where citizens do not regard the state as entirely benign, where trust has been violated, attending only to the narrowly technical aspects of childbirth cannot guarantee the increased popularity of institutional deliveries. Put another way: delay in seeking treatment is only *temporally* prior to the second and third phases of delay. Perceptions of the problems associated with institutional care—whether technical or interpersonal—may themselves delay the quest for treatment (cf. George et al., 2005; HRW, 2009; Singh et al., 2004). Talking of ‘delays’ in seeking institutional care can easily become victim-blaming, in which villagers are portrayed as foolish or casual. But there were compelling reasons why Razia remained at home so long despite the warnings about her safety, and why choosing between government and private care was fraught: a lethal mix of Rashid’s poverty, the Dharmnagri ANM’s unavailability, and mistrust of government health care services and staff more generally.

Improving the accountability of front-line Government health staff is not easy, however. As in Jhakri, most patients respond by withdrawal (or ‘exit’) from government services rather than raising their voices to complain. The poor feel vulnerable to repercussions, and actually following a complaint through to a conclusion is costly and time-consuming. Front-line staff themselves are at the bottom of a power hierarchy and can rarely change the systemic conditions that provide them with inadequate resources and support for women needing emergency obstetric care. Improved training procedures, incentive structures that reward staff who are honest and polite as well as competent, and legal procedures to deal with corrupt or abusive staff (such as Public Interest Litigation sponsored by civil society organisations) would all make a difference. As yet, though, there is no sign that such issues are being taken seriously. Unlike erecting new buildings and buying in more drugs, such shifts require the dismantling of a long-standing political economy of health care provision. The most poverty-stricken and powerless members of Indian society experience deep-seated class, caste and urban prejudices against them in many contexts, including when they seek health care. Encouraging institutional deliveries without rectifying these serious shortcomings in the modes of operation within government services is a seriously flawed approach to reducing maternal morality. There is a long-term credibility gap: persuading villagers of the superiority of institutional deliveries will be an uphill task.
